# PU.1 Is Essential for CD11c Expression in CD8^+^/CD8^−^ Lymphoid and Monocyte-Derived Dendritic Cells during GM-CSF or FLT3L-Induced Differentiation

**DOI:** 10.1371/journal.pone.0052141

**Published:** 2012-12-20

**Authors:** Xue-Jun Zhu, Zhong-Fa Yang, Yaoyu Chen, Junling Wang, Alan G. Rosmarin

**Affiliations:** 1 Cell and Molecular Biology Laboratory, Division of Hematology, Department of Medicine, Jiangsu Provincial Traditional Chinese Medical Hospital, Nanjing, China; 2 Division of Hematology-Oncology, Department of Medicine, University of Massachusetts Medical School, Worcester, Massachusetts, United States of America; University of Bergen, Norway

## Abstract

Dendritic cells (DCs) regulate innate and acquired immunity through their roles as antigen-presenting cells. Specific subsets of mature DCs, including monocyte-derived and lymphoid-derived DCs, can be distinguished based on distinct immunophenotypes and functional properties. The leukocyte integrin, CD11c, is considered a specific marker for DCs and it is expressed by all DC subsets. We created a strain of mice in which DCs and their progenitors could be lineage traced based on activity of the CD11c proximal promoter. Surprisingly, we observed levels of CD11c promoter activity that were similar in DCs and in other mature leukocytes, including monocytes, granulocytes, and lymphocytes. We sought to identify DNA elements and transcription factors that regulate DC-associated expression of CD11c. The ets transcription factor, PU.1, is a key regulator of DC development, and expression of PU.1 varies in different DC subsets. GM-CSF increased monocyte-derived DCs in mice and from mouse bone marrow cultured in vitro, but it did not increase CD8^+^ lymphoid-derived DCs or B220^+^ plasmacytoid DCs. FLT3L increased both monocyte-derived DCs and lymphoid-derived DCs from mouse bone marrow cultured in vitro. GM-CSF increased the 5.3 Kb CD11c proximal promoter activity in monocyte-derived DCs and CD8^+^ lymphoid-derived DCs, but not in B220^+^ plasmacytoid DCs. In contrast, FLT3L increased the CD11c proximal promoter activity in both monocyte-derived DCs and B220^+^ plasmacytoid DCs. We used shRNA gene knockdown and chromatin immunoprecipitation to demonstrate that PU.1 is required for the effects of GM-CSF or FLT3L on monocyte-derived DCs. We conclude that both GM-CSF and FLT3L act through PU.1 to activate the 5.3 Kb CD11c proximal promoter in DCs and to induce differentiation of monocyte-derived DCs. We also confirm that the CD11c proximal promoter is not sufficient to direct lineage specificity of CD11c expression, and that additional DNA elements are required for lineage-specific CD11c expression.

## Introduction

Dendritic cells (DCs) are bone marrow-derived cells that play crucial roles in regulating and integrating innate and adaptive immune responses. DCs sample the environment, recognize invading pathogens through pattern recognition Toll-like receptors (TLRs), and initiate protective T cell responses by presenting antigens to lymphocytes. Because of their highly developed antigen presenting capacity, DCs have drawn attention for use in cell therapy [1], [2]. DCs are a heterogeneous group of cells that have been classified into distinct subsets, primarily based on patterns of cell surface antigen expression. The integrin αL chain, CD11c, is considered relatively specific for DCs. CD11c is expressed on all mature DC subsets in mouse, and its expression increases as DCs mature from DC progenitors. Subsets of mature DCs in blood and the lymphatic system include conventional DCs (cDCs; CD11c^+^CD4^−^CD8^+^CD11b^−^MHCII^+^, or CD11c^+^CD4^+^CD8^−^CD11b^−^MHCII^+^), plasmacytoid DCs (pDCs; CD11c^+^B220^+^Ly6C^+^), and monocyte-derived DCs (CD11c^+^CD11b^+^CD8^−^CD4^−^MHCII^+^) [3], [4], [5].

Differentiation of DCs from hematopoietic progenitor cells depends on the activity of cytokines, including FLT3L (FMS-related tyrosine kinase 3 ligand) and GM-CSF (granulocyte-monocyte colony stimulating factor). Mouse hematopoietic progenitor cells cultured with FLT3L generate all cDC subsets and pDCs [6], [7], and mice deficient for Flt3l have markedly reduced numbers of cDCs and pDCs [8]. GM-CSF stimulates cultured bone marrow cells to differentiate into monocyte-derived DCs [9], [10], but GM-CSF is not essential for DC development in the steady state, as mice lacking the GM-CSF receptor (GM-CSFR) have only slightly reduced numbers of DCs [11].

DC development also depends on transcription factors that mediate extracellular cues from cytokines and their cognate receptors. For example, interferon regulatory factor 8 (IRF-8) is essential for the development of CD8^+^ cDCs and pDCs [12], [13], while IRF-4 deficiency in mice is associated with a marked reduction in CD8^−^CD4^+^ cDCs [14], [15]. The E protein, E2-2, controls DC progenitor diversion to pDCs, as it is abundantly expressed by pDCs and is required for pDC lineage specification [16], [17]. Conditional deletion in the hematopoietic compartment of the ets transcription factor, PU.1, blocks development of both cDC and pDC subsets [18]. PU.1 expression is high in early DC progenitors and cDCs, but remains low in pDCs [3], [4]. PU.1 controls the expression of FLT3 and GM-CSFR on progenitor cells, but the mechanism by which it regulates DC lineage fate choice remains to be elucidated.

In this study, we used lineage fate mapping to examine activity of the CD11c proximal promoter under steady state conditions, and in response to GM-CSF induction *in vivo* and *ex vivo*. Although CD11c is considered relatively specific for DCs, we found that the 5.3 Kb CD11c proximal promoter is active in about 30% cells of myeloid and lymphoid cells from bone marrow, spleen and peripheral blood. Unexpectedly, CD11c promoter activity was only slightly higher in DCs than in other hematopoietic lineages. Growth of mouse bone marrow cells in the presence of GM-CSF or FLT3L induced monocyte-derived DC differentiation, and stimulated CD11c promoter activity in nearly 90% of DCs. Similarly, treatment of mice with GM-CSF markedly increased activity of the CD11c proximal promoter in both CD8a^+^ and CD8a^−^ cDCs in spleen, but not in B220^+^ pDC subsets or other hematopoietic lineages. Chromatin immunoprecipitation (ChIP) demonstrated that PU.1 and IRF-4, which are known to be essential for DC development, bound DNA elements within the 5.3 kb CD11c proximal promoter. Expression of these transcription factors increased in response to GM-CSF or FLT3L-induced DC differentiation. shRNA-mediated knock down of PU.1 in cultured bone marrow cells inhibited GM-CSF or FLT3L-induced monocyte-derived DC differentiation and reduced CD11c promoter activity in response to these cytokines. In mouse spleen, expression of Irf-4 is maintained at high level only in CD8a^−^CD4^+^ cDCs. Thus, GM-CSF or FLT3L activates the 5.3 kb CD11c proximal promoter through synergy with essential DC transcription factors, including PU.1 and IRF-4, whose expression are also induced by GM-CSF or FLT3L signaling during differentiation of cultured monocyte-derived DCs. Although administration of GM-CSF in mice did not promote differentiation of CD8^+^ cDCs in spleen, it was sufficient to CD11c promoter activity in both CD8^+^ and CD8^−^ cDCs, most likely through the up-regulation of PU.1 in these cells. This suggests that additional promoter or enhancer elements, together with other transcription factors, account for the CD11c expression in B220^+^ pDC subsets in spleen, and its lineage-specific expression pattern exclusively in DCs.

## Results

### The CD11c Proximal Promoter Controlled GFP Expression cannot be Detected in the Majority of DC Subsets from the CD11c-Cre Mice

CD11c is considered a relatively specific marker of DCs, and CD11c promoter-driven expression of Cre recombinase has been used to inactivate genes in DCs. Stranges, *et al.* used a 5.3 kb genomic DNA fragment that includes the mouse CD11c proximal promoter to drive expression of both Cre recombinase and enhanced green fluorescent protein (GFP); because GFP is co-transcribed with Cre from the CD11c promoter, the intensity of green fluorescence should indicate the specificity and efficacy of the CD11c promoter. Among several transgenic mouse strains, strain 4097 exhibited the highest level of GFP in CD11c^+^ DCs [19]. However, even in CD11c-Cre mouse strain 4097, the GFP expression in lymph node CD11c^+^ DCs was not markedly higher than non-transgenic littermate controls, so that there was a substantial portion of GFP^−^CD11c^+^ DCs from the CD11c-Cre mice that overlapped with the GFP^−^CD11c^+^ DCs from the non-transgenic littermate control mice. Therefore, the efficacy and specificity of the CD11c-Cre transgene in the CD11c-Cre mouse strain 4097 require better evaluation.

**Figure 1:**
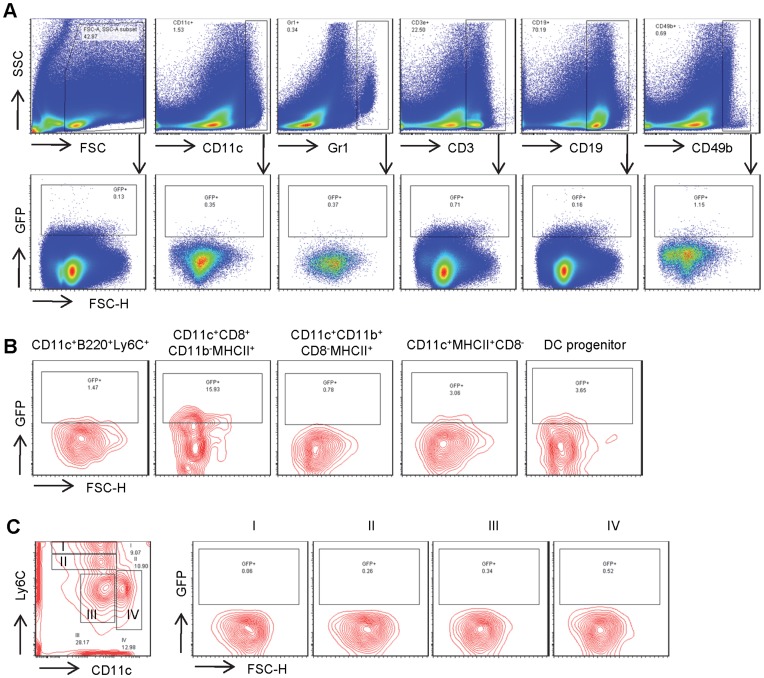
CD11c-Cre mice have minimal GFP^+^ DCs in spleen. Flow cytometric analysis of CD11c-Cre mouse spleen cells for their GFP expression in **A**) DCs, myeloid cells, T- and B-lymphocytes and natural killer (NK) cells. **B**) Flow cytometric analysis of the same mouse spleen cells for GFP expression in different DC subsets including pDCs (CD11c^+^B220^+^CD8^−^CD11b^−^Ly6C^+^), CD8^+^ DCs (CD11c^+^CD8^+^CD11b^−^MHCII^+^), CD8^−^CD11b^+^ DCs (CD11c^+^CD8^−^CD11b^+^MHCII^+^), inactivated (CD11c^+^MHCII^+^CD8^+^) and activated DCs (CD11c^+^MHCII^+^CD8^−^) and DC progenitors (Lineage^−^CD11c^+^CD11b^−^B220^−^CD43^+^). **C**) Analysis of CD11b^+^ spleen cells for GFP expression during their maturation from group I (CD11c^−^Ly6C^hi^), group II (CD11c^−^Ly6C^low^), to group III (CD11c^−^Ly6C^−^), and group IV (CD11c^+^Ly6C^−^).

We analyzed GFP expression in DC and non-DC subsets of cells from spleen, blood, and bone marrow of CD11c-Cre GFP mouse train 4097; littermates that lacked CD11c-Cre GFP served as negative controls. The percentage of GFP^+^ cells in total spleen cells was less than 1% (Figure 1A). Because expression of GFP reflects CD11c promoter activity, one would expect the percentage of GFP^+^ cells to be higher in CD11c^+^ DC subsets than in the total cell population or in other cell types. The percentage of GFP^+^ cells in various CD11c^−^ populations, including myeloid cells (Gr1^+^), T-lymphocytes (CD3^+^), B-lymphocytes (CD19^+^), and natural killer (NK) cells (CD49b^+^) varied from 0.2% to 1.1% (Figure 1A); thus, the percentage of GFP^+^ cells among these other cell types was not significantly different from the percentage of GFP^+^ cells in CD11c^+^ cells in spleen (0.4%). Stranges et. al. only characterized DCs with the single surface marker CD11c [19]. It is possible that the portion of GFP-expressing DCs was underestimated, and that better defined DC populations with additional surface antigens (B220 and Ly6C for pDCs; CD8, CD11b and MHC class II (MHCII) for cDCs) could significantly increase the percentages of GFP-expressing cells. Although CD8^+^ cDCs (CD11c^+^CD8^+^CD11b^−^MHCII^+^) exhibited marked increase in the percentage of GFP^+^ cells (16%), the majority of CD8^+^ cDCs were still GFP^−^. Furthermore, neither the percentages of GFP^+^ cells nor the GFP level was significantly higher than non-DC cells in spleen pDCs (CD11c^+^B220^+^Ly6C^+^), CD8^−^ cDCs (CD11c^+^CD8^−^CD11b^+^MHCII^+^), MHCII^+^ activated DCs (CD11c^+^MHCII^+^CD8^−^), or in bone marrow derived DC progenitors (Lineage^−^CD11c^+^CD11b^−^B220^−^CD43^+^) (Figure 1B).

Expression of CD11c increases and Ly6C decreases as DCs mature. Based on expression of these antigens, splenic CD8^−^CD11c^+^ DCs can be divided into groups I, II, III, and IV, which represent increasing states of differentiation [20]. Because CD11c expression increases during DC maturation, the percentage of GFP^+^ cells should be expected to increase. However, we observed no significant increase in the percentage of GFP^+^ cells in group I compared to groups II, III, or IV (Figure 1C). GFP expression was detected in only a small fraction of the CD11c^+^ DCs, and some CD11c^−^ cells contain comparable percentage of GFP^+^ cells. We conclude that expression of CD11c-GFP expression is not specific for CD11c^+^ DCs, and the CD11c proximal promoter did not accurately recapitulate the expression of native CD11c in mouse DC subsets.

### Generation of Mice Bearing both CD11c-Cre and Rosa26 loxP-STOP-loxP

**Figure 2:**
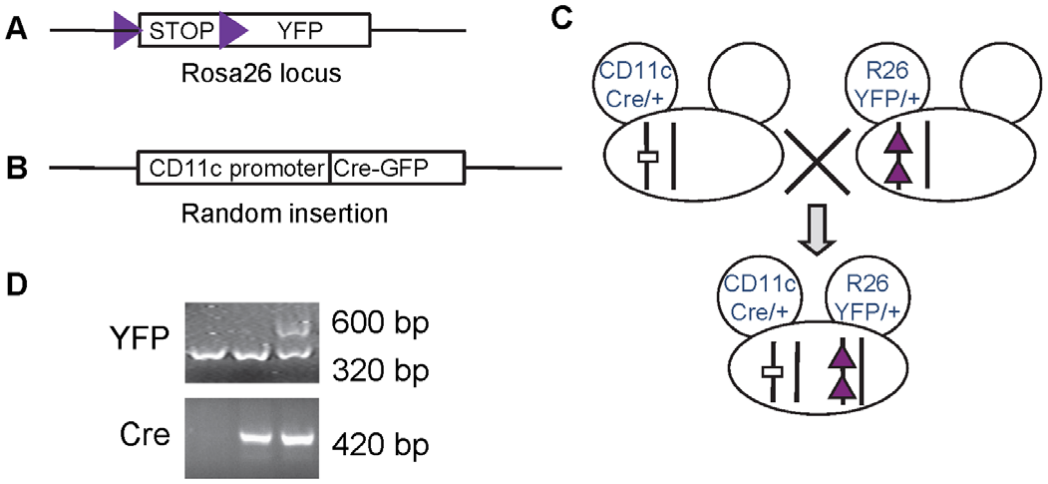
Generation of mice bearing both CD11c-Cre and Rosa26 loxP-STOP-loxP YFP. Diagrams show **A**) the construct of loxP (triangles) flanked transcription stop signal sequence (SA) followed by YFP under the control of Rosa26 locus; and **B**) the 5.3 kb CD11c proximal promoter controlled Cre recombinase followed by IRES and GFP. **C**) the diagram demonstrates the breeding strategy of generating mice carrying both CD11c-Cre and Rosa26 loxP-STOP-loxP. **D**) PCR on genomic DNA from offspring mice of the breeding pairs in C. Mice carrying both CD11c-Cre and Rosa26 loxP-STOP-loxP demonstrate both the 420 bp Cre product and the 600 bp YFP product.

Because CD11c proximal promoter activity (i.e. CD11c-Cre-GFP expression) did not reflect expression of native CD11c in DCs, we utilized CD11c promoter-driven Cre recombinase expression to lineage trace DCs. The similar strategy has been used to examine gene activation/deletion restricted in DCs of another CD11c-Cre transgenic mouse train [20]. We bred CD11c-Cre mice to a mouse strain that bears the enhanced yellow fluorescent protein (YFP) gene that was knocked-into a locus immediately downstream of the endogenous Rosa26 gene promoter and loxP-STOP-loxP sequences [20], [21], as illustrated in Figure 2A, B, C. Rosa26 is expressed in all tissues, and flow cytometry can be used to detect YFP expression that is activated by Cre-mediated deletion of the STOP sequence. Mice carrying both transgenes genes were identified by PCR on their genomic DNA for the presence of CD11c-Cre construct (420 bps) and the Rosa26 loxP-STOP-loxP (600 bps) (Figure 2D).

### YFP Expression occurs in all Lineages of Hematopoietic Cells from Mice Bearing CD11c-Cre and Rosa26 loxP-STOP-loxP

**Figure 3:**
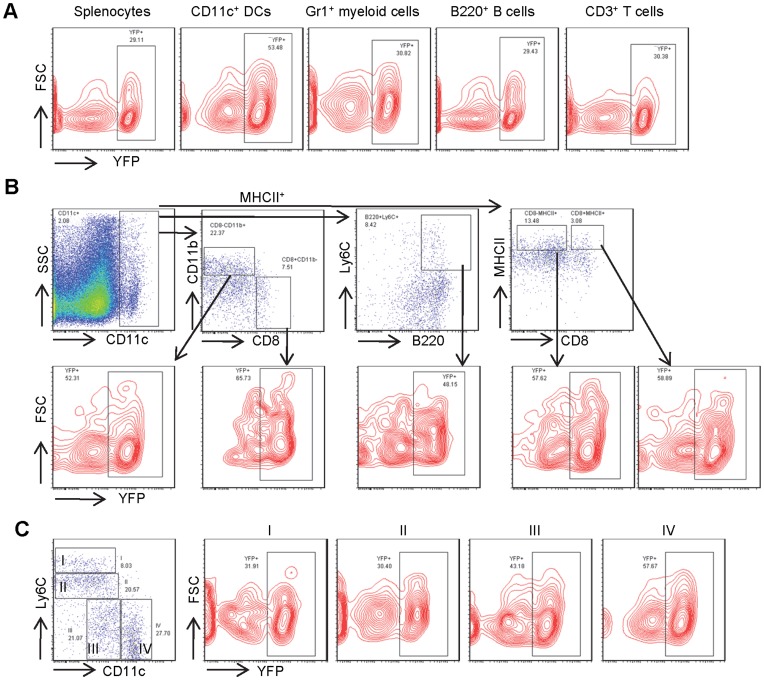
CD11c-Cre mediated recombination to activate YFP expression in spleen cells. Flow cytometric analysis of spleen cells from mice bearing both CD11c-Cre and Rosa26 loxP-STOP-loxP for the Cre activated YFP expression in **A**) various lineages including CD11c^+^ DCs, Gr1^+^ myeloid cells, B220^+^ B- and CD3^+^ T-lymphocytes, and in **B**) DC subsets including CD8^+^ DCs, CD8^−^CD11b^+^ DCs, pDCs, inactivated and activated DCs. **C**) Analysis of YFP expression in CD11b^+^ DCs during their maturation from group I to group IV.

As illustrated in Figure 3A, YFP expression was observed in about 30% of total spleen cells and 26% of peripheral blood leukocytes. Because each of these hematopoietic organs contains only a small percentage of CD11c^+^ DCs, the substantially higher percentages of YFP^+^ cells indicates that CD11c-Cre must also be active in non-DC lineages. In fact, approximately 30% of Gr1^+^ granulocytes, B220^+^ B-lymphocytes, and CD3^+^ T-lymphocytes in spleen express YFP (Figure 3A). Unexpectedly, nearly half of CD11c^+^ DCs were YFP negative. Analysis of various DC subsets with additional cell surface markers demonstrated significant increases of YFP^+^ cells in CD8^+^ cDCs (CD11c^+^CD8^+^CD11b^−^MHCII^+^) (65%), CD8^−^ cDCs (CD11c^+^CD8^−^CD11b^+^MHCII^+^) (52%), pDCs (CD11c^+^B220^+^Ly6C^+^) (48%), inactivated (CD11c^+^CD8^+^MHCII^+^) (59%) and activated DCs (CD11c^+^CD8^−^MHCII^+^) (58%). But none of their percentages of YFP^+^ DCs were even close to 100% (Figure 3B). CD11c expression increases during the maturation of spleen DCs from stage I through II and III to stage IV and, as expected, the percentage of YFP^+^ DCs increased from 32% to 58% as DCs differentiated from stage I through stage IV (Figure 3C), however, even the most mature DCs, i.e. stage IV, were not 100% YFP positive. This finding demonstrates that CD11c proximal promoter-driven Cre-mediated recombination occurs in all hematopoietic lineages. Although there was higher percentage of YFP^+^ cells in CD11c^+^ DCs, not all CD11c^+^ DCs were YFP^+^. This finding is in great contrast to the observation from Boris Reizis group, where in a different strain of CD11c-Cre transgenic mouse, the percentages of YFP^+^ cells ranges from 86% to 97% in DC subsets, and were from 0.3% to 12% in nonDC cells [20]. Thus, the 5.3 Kb CD11c proximal promoter activity is neither fully sensitive nor specific for detection of DCs.

### YFP Expression Occurs in Hematopoietic Stem and Progenitor Cells from Mice Bearing CD11c-Cre and Rosa26 loxP-STOP-loxP

**Figure 4:**
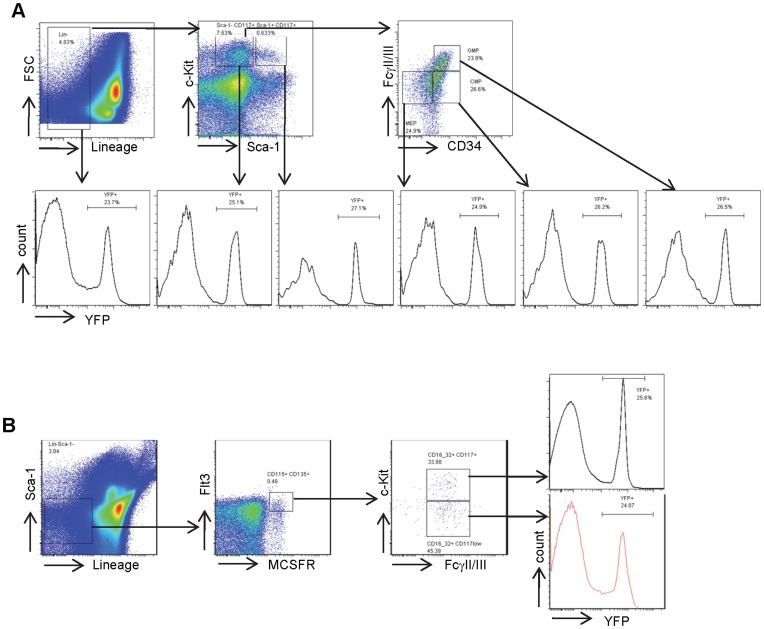
CD11c-Cre mediated recombination to activate YFP expression in mouse bone marrow hematopoietic stem and progenitor cells. Flow cytometric analysis of bone marrow cells from mice bearing both CD11c-Cre and Rosa26 loxP-STOP-loxP for YFP expression in **A**) lineage negative cells, early myeloid progenitors (LK), stem cells (LSK) and CMPs, GMPs, MEPs; and in **B**) DC progenitor cells including MDPs and CDPs.

Bone marrow derived DC progenitors express low levels of CD11c, but hematopoietic stem cells (HSCs), myeloid, and lymphoid progenitor cells do not express CD11c [22]. If expression of the CD11c-Cre transgene mimics expression of endogenous CD11c, lineage negative (Lin^−^) stem and progenitor cells should be CD11c^−^ and YFP^−^. Surprisingly, nearly a quarter of Lin- bone marrow cells expressed YFP (Figure 4A and 4B). Furthermore, more than one quarter of HSCs (Lin^−^ c-Kit^+^ and Sca1^+^, LSK) and myeloid progenitor cells (Lin^−^ c-Kit^+^ Sca1^−^, LK) expressed YFP (Figure 4A). Bone marrow DC progenitor cells that are Lin^−^, Sca1^−^, Flt3^+^ and Mcsfr^+^, including MDP (c-Kit^+^FcγII/III^+^) and CDP (c-Kit^−^FcγII/III^+^), demonstrated similar percentages of YFP^+^ cells (Figure 4B). Thus, the 5.3 Kb CD11c proximal promoter is active under steady state conditions in a significant portion of HSCs and progenitor cells.

### GM-CSF and FLT3L Induced a Significant Increase in Transgenic 5.3 Kb CD11c Proximal Promoter Activity in Cultured Bone Marrow Derived DC Subsets

**Figure 5:**
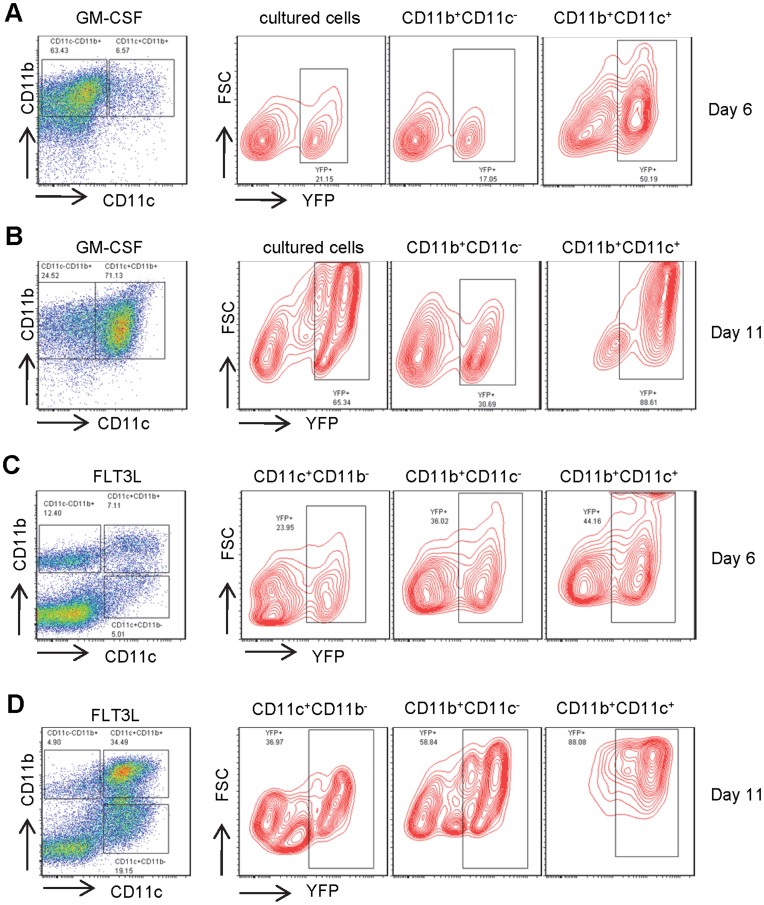
CD11c-Cre mediated recombination to activate YFP expression in mouse bone marrow cells cultured with GM-CSF or FLT3L. Flow cytometric analysis of bone marrow cells from mice bearing both CD11c-Cre and Rosa26 loxP-STOP-loxP for their YFP expression in CD11c^+^ and CD11c^−^ cells after cultured with GM-CSF or FLT3L for 6 days **A**) and **C**), and for 11 days **B**) and **D**), respectively.

Cytokine signaling plays crucial roles in DC development. GM-CSF drives the formation of DCs from cultured mouse bone marrow cells or human peripheral monocytes [9], [10], and GM-CSF promotes monocyte derived DC development under inflammatory conditions [9]–[11]. FLT3L supports the development of DCs *in vitro* [7], [8], [23], and FLT3L signaling is required for *in vivo* steady-state development of various mouse DC subsets [8]. To test whether the 5.3 Kb CD11c proximal promoter is responsive to GM-CSF or FLT3L signaling, bone marrow cells from mice bearing CD11c-Cre and Rosa26 loxP-STOP-loxP were cultured *in vitro* with GM-CSF or FLT3L to induce DC differentiation. After six days growth in GM-CSF, the majority of the bone marrow cells were CD11b^+^, and CD11c^+^ cells increased to 7% of total cultured bone marrow cells (Figure 5A). The percentage of YFP^+^ cells in CD11b^+^CD11c^+^ DCs was 50%, which was markedly higher than that in total cultured cells or CD11b^+^CD11c^−^ non-DC cells (Figure 5A). The percentage of CD11c^+^ cells increased further to 71% after eleven days of growth in GM-CSF (Figure 5B). Both the intensity of YFP expression and the percentage of YFP^+^ CD11c^+^CD11b^+^ DCs increased from 50% on day 6 to nearly 90% on day 11 (Figure 5B). In FLT3L treated cell culture, however, the CD11b^+^ cells were less than 20%, and the CD11c^+^ cells increased to more than 12% of total cultured bone marrow cells after six days induction by FLT3L. Consistent with previous reports, FLT3L induced an additional population of CD11c^+^CD11b^−^B220^+^ pDCs (Figure 5C) [23]. Similarly, the percentage of YFP^+^ cells in FLT3L treated CD11b^+^CD11c^+^ DCs was 44%, which was higher than that in CD11c^+^CD11b^−^B220^+^ pDCs or CD11b^+^CD11c^−^ non-DC cells (Figure 5C). Once again, the percentage of CD11c^+^ cells increased further to 54% after eleven days of treatment of FLT3L (Figure 5D). Both the intensity of YFP expression and the percentage of YFP^+^ DCs increased from 44% on day 6 to nearly 90% on day 11. The YFP^+^ CD11c^+^CD11b^−^ pDCs increased from 36% on day 6 to 58% on day 11 (Figure 5D). Thus, the 5.3 Kb CD11c proximal promoter is highly responsive to both GM-CSF and FLT3L signaling during DC development in cultured mouse bone marrow cells.

### GM-CSF Activated the Transgenic 5.3 Kb CD11c Proximal Promoter in CD8^+^ and CD8^−^ cDCs, but not pDCs, During the Induced Differentiation of DCs in Mouse Spleen

**Figure 6:**
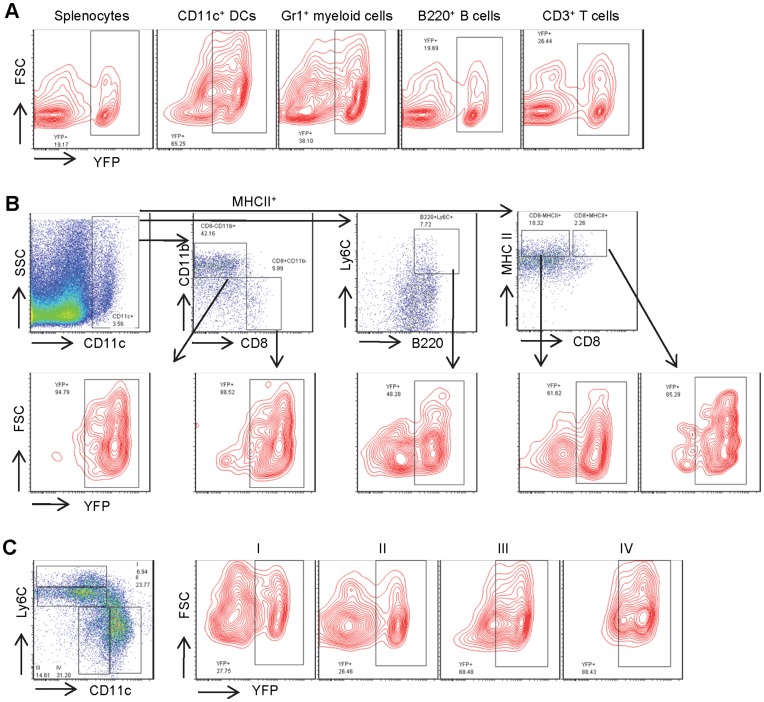
CD11c-Cre mediated recombination to activate YFP expression in spleen cells after GM-CSF administration in mice. Mice bearing both CD11c^−^Cre and Rosa26 loxP-STOP-loxP were injected with GM-CSF followed by flow cytometric analysis of their spleen cells for YFP expression in **A**) different lineages including DCs, myeloid cells, B- and T-lymphocytes, and in **B**) DC subsets including CD8^+^ DCs, CD8^−^CD11b^+^ DCs and pDCs. **C**) Analysis of YFP expression in CD11b^+^ DCs during their maturation from group I to group IV.

GM-CSF stimulates DC differentiation in culture, but it is not essential for the steady-state DC differentiation *in vivo* [11]. GM-CSF has a greater role in production of monocyte-derived DCs than in other DC subsets. To test whether GM-CSF can activate the 5.3 Kb CD11c proximal promoter *in vivo*, CD11c-Cre and Rosa26 loxP-STOP-loxP mice were injected with GM-CSF, or saline control. Flow cytometric analysis of cells harvested 48 hours after injection demonstrated that saline injection did not change the percentage of YFP^+^ spleen cells in DCs, myeloid or lymphoid cells, and the percentages of the YFP^+^ cells were comparable to uninjected mice in Figure 3 (data not shown). As shown in Figure 6A, injection of GM-CSF significantly increased the percentage and the total number of CD11c^+^ DCs in spleen. There was a significant increase in the percentage of YFP^+^ cells in CD11c^+^ DCs, a slight increase in Gr1^+^ myeloid cells, and a mild decrease in B220^+^ B-lymphocytes (Figure 6A). As shown in Figure 6B, the CD11c^+^CD11b^+^CD8^−^MHCII^+^ cDCs represented the majority of the increased DCs stimulated by GM-CSF in total spleen cells. Similar to the observation in cultured cells, mice injected with GM-CSF exhibited a substantial increase in the percentage of CD8^−^CD11b^+^ DCs in spleen and increased the percentage of YFP^+^ cells among this population (compare Figure 6B to Figure 3B). GM-CSF administration did not increase the percentage of CD11c^+^CD11b^−^CD8^+^MHCII^+^ cDCs, CD11c^+^CD8^+^MHCII^+^ inactiveated or CD11c^+^CD8^−^MHCII^+^ activated DCs, but it did markedly increased the percentage of YFP^+^ cells among these splenic DC subsets (Figure 6B). There was no increase either in the percentage of CD11c^+^B220^+^pLy6C^+^ pDCs, or in the percentage that were YFP^+^ (Figure 6B). In addition, the most mature CD11b^+^Ly6C^−^CD11c^hi^ DCs in spleen demonstrated nearly 90% YFP^+^ cells, as shown in Figure 6C. Thus, the 5.3 Kb CD11c proximal promoter, as measured by YFP activity, is highly responsive to GM-CSF in both CD8^−^CD11b^+^ and CD8^+^CD11b^−^ cDCs during GM-CSF induced DC differentiation *in vivo*, but not in pDCs.

### Cis-regulatory Elements on 5.3 Kb CD11c Proximal Promoter Contain Motifs that are Bound by Crucial DC Transcription factors During Cytokine Induced Differentiation

**Figure 7:**
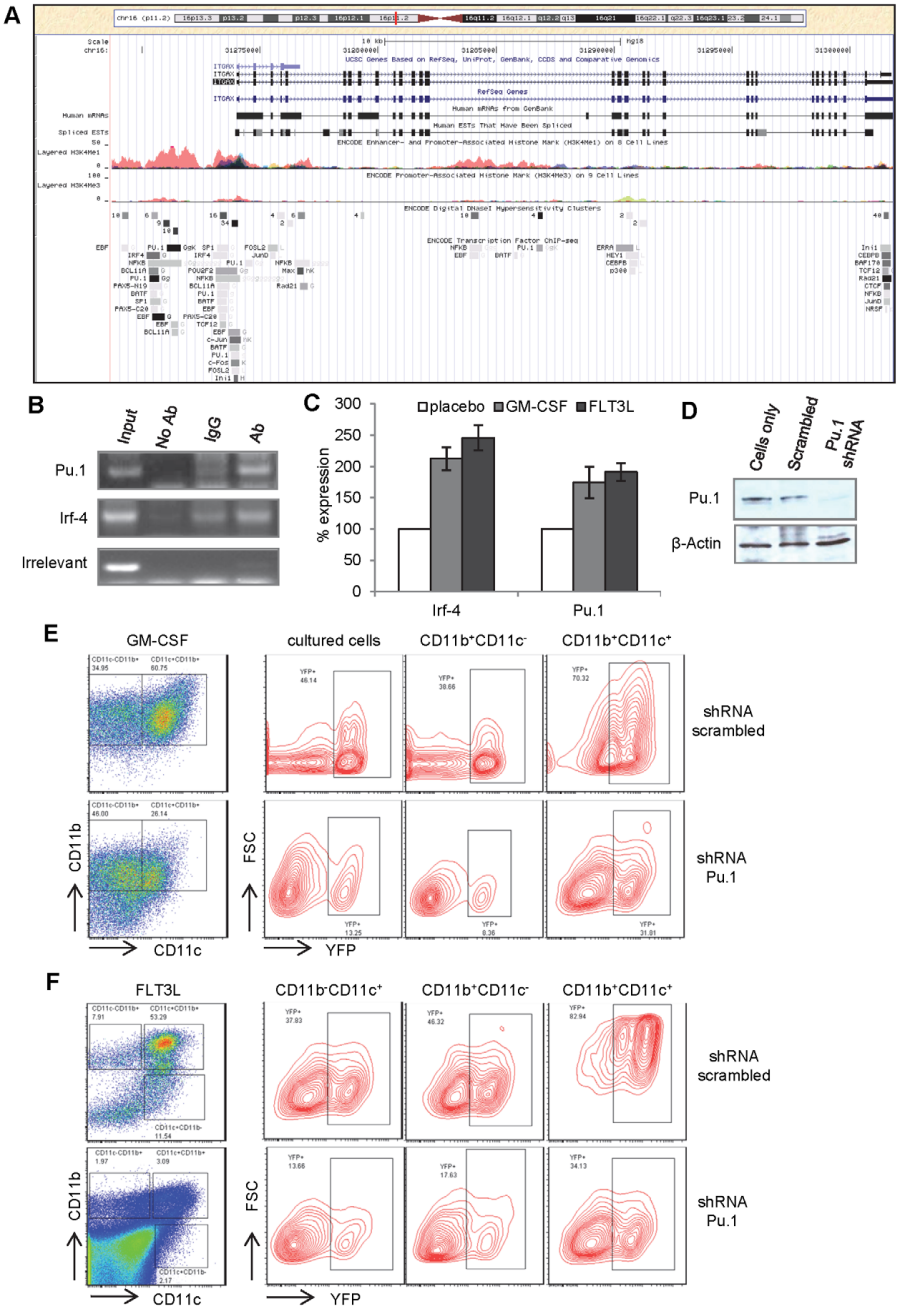
CD11c proximal promoter contains binding sites for PU.1 and IRF-4, which are essential for GM-CSF or FLT3L-induced CD11c activation. **A**) Diagram demonstrating the human 5.3 kb CD11c proximal promoter. Transcription factors binding to this region according to ChIP-seq analysis of existing data are illustrated in the diagram. **B**) ChIP assays on cultured mouse bone marrow cells with GM-CSF for the binding of Pu.1 and Irf-4 to the CD11c proximal promoter region. **C**) Quantitative PCR on reverse transcribed mRNA from mouse bone marrow cells cultured with GM-CSF or FLT3L to evaluate expression of Pu.1 and Irf-4. Bone marrow cells from mice bearing both CD11c-Cre and Rosa26 loxP-STOP-loxP were transduced with retrovirus carrying either scrambled control shRNA or shRNA against Pu.1 followed by puromycin selection of transduced cells. Immunoblotting **D**) and Flow cytometric analysis of the transduced cells for the expression YFP in both CD11c^+^ and CD11c^−^ cells 11 days after culturing with GM-CSF **E**), or FLT3L F).

The increased percentage of YFP^+^ DCs suggests that the 5.3 Kb CD11c proximal promoter contains cis-elements that are responsive to GM-CSF and FLT3L signaling. Alignment of a 5.3 kb DNA sequence upstream of the CD11c transcription start site (TSS) among mammalian species identified an evolutionarily conserved region with about 2 kb that spans the TSS (Figure 7A). Chromatin immunoprecipitation (ChIP) followed by high throughput deep sequencing (ChIP-seq) on cell lines revealed potential transcription factor binding sites in this conserved promoter region (Figure 7A), including IRF-4 and PU.1, both of which are known to be crucial for the development of DCs, B-lymphocytes and myeloid cells.

Bone marrow cells were cultured *in vitro* with GM-CSF or FLT3L for 7 days and analyzed by ChIP assay. As shown in Figure 7B, both Irf-4 and Pu.1 bound the region of the CD11c proximal promoter 2 kb upstream of the TSS in response to GM-CSF (Figure 7B) or FLT3L (data not shown). Furthermore, the mRNA levels of Irf-4 and Pu.1 in bone marrow cells cultured with GM-CSF or FLT3L increased up to two fold, compared to the same cells in the absence of GM-CSF or FLT3L (Figure 7C). Irf-4 expression is high only in CD8^−^CD4^+^ cDCs, and is essential for their development. PU.1 expression is high in both DC progenitors and mature cDCs, but low in pDCs. Coincidentally, GM-CSF-induced activation of the 5.3 Kb CD11c proximal promoter occurs in CD8^+^ and CD8^−^CD11b^+^ cDCs, but not in pDCs. This suggests that GM-CSF-induced Pu.1 expression in cDCs is required for activation of CD11c proximal promoter. To test this hypothesis, bone marrow cells were transduced with retrovirus containing shRNA specifically against Pu.1 or scrambled control shRNA. Immunoblotting on protein extracts from cultured bone marrow cells demonstrated the near complete loss of Pu.1 protein expression suppressed by shRNA specifically against Pu.1 gene, but not by scrambled control shRNA (Figure 7D). As shown in Figure 7E–7F, Pu.1 knock-down markedly inhibited GM-CSF (Figure 7E) or FLT3L-induced (Figure 7F) differentiation of CD11c^+^ monocyte-derived DCs and greatly reduced the percentage of YFP^+^ DCs, compared to control cells transduced with scrambled shRNA. Pu.1 knockdown also reduced FLT3L-induced differentiation of pDCs and the percentage of YFP^+^ pDCs (Figure 7F). To summarize, the 5.3 Kb CD11c proximal promoter contains Irf-4 and Pu.1 binding elements that are responsive to GM-CSF or FLT3L-induced DC differentiation *in vivo* and *ex vivo*, Irf-4 and Pu.1 expression increase in response to GM-CSF treatment, and Pu.1 is essential for both GM-CSF and FLT3L-induced CD11c expression in monocyte-derived DCs in culture. Thus, Pu.1 appears to be the key regulator that controls CD11c expression in both CD8^+^ and CD8^−^ lymphoid resident cDCs.

## Discussion

The leukocyte integrin, CD11c, is expressed by DCs and is considered to be a relatively specific marker of this lineage. In this study we demonstrated that expression of GFP driven by the 5.3 kb proximal promoter of mouse CD11c did not accurately reflect expression of native CD11c. We generated mice that are transgenic for both CD11c-Cre and Rosa26-loxP-STOP-loxP YFP, which can trace activation of the CD11c proximal promoter during DC differentiation. We found that the CD11c promoter was also active in HSCs, hematopoietic progenitor cells, and in all categories of mature leukocytes. Thus, the CD11c proximal promoter is neither sufficient nor specific for driving CD11c expression in DCs, and we conclude that additional promoter or enhancer regions of CD11c must be required to control its specific expression pattern in DCs. Treatment of bone marrow cells *in vitro* with GM-CSF or FLT3L increased CD11c expression in monocyte-derived (CD11b^+^) DCs. *In vivo*, GM-CSF activated the CD11c promoter in CD11b^+^ DCs and in CD8^+^ DCs, but not in B220^+^ pDCs. We showed that the transcription factors, Irf-4 and Pu.1, bound to the CD11c proximal promoter, and that Pu.1 knock down reduced GM-CSF or FLT3L-induced CD11c promoter activation. Together with the observation that expression of Pu.1 is high in CD8^+^ and CD11b^+^ cDCs, but low in B220^+^ pDCs, we conclude that high level expression of Pu.1 is essential for the control of CD11c expression in CD11b^+^ and in CD8^+^ cDCs.

The *CD11c* gene encodes the integrin αL chain, a leukocyte cell surface antigen that is routinely used as a specific marker of DCs, but the cis-regulatory elements that restrict its expression to DCs have not been identified. We used two different approaches to examine CD11c promoter activity in mouse DCs and other blood cells. Expression of GFP directly reflects CD11c promoter activity at the time of analysis. In contrast, Cre activation of YFP expression reflects a cumulative effect of CD11c activity during the development of particular lineages. Expression of YFP by HSCs and progenitors indicates that the CD11c promoter is active in immature hematopoietic cells. Thus, Cre activation of YFP reflects the derivation of hematopoietic cells. It may also serve as a more sensitive indicator of CD11c promoter activity than the GFP model.

Growth and differentiation of DCs depend on signals from extracellular growth factors, including GM-CSF and FLT3L. The transcription factors, IRF-4 and PU.1, are critical for development of DCs, *i.e.* PU.1 is required for the development of all DC subsets, while IRF-4 is essential for CD8^−^CD4^+^ lymphoid resident cDCs in spleen. Our findings demonstrated that expression of transcription factors PU.1 and IRF-4 is induced by GM-CSF or FLT3L. Expression of CD11c, the DC specific target gene of both Pu.1 and Irf-4, is rapidly increased in response to GM-CSF or FLT3L induction in mouse bone marrow cells. This demonstrates that the coordination of extrinsic GM-CSF or FLT3L signal with the transcriptional programming by PU.1 and IRF-4 in DCs contributes to the DC specific gene expression and committed DC differentiation.

GM-CSF induces development of monocyte-derived DCs in culture, while FLT3L induces the differentiation of both monocyte-derived and lymphocyte-derived DCs [8]. We showed that monocyte-derived DCs increased significantly in GM-CSF-treated mice. It appears that PU.1 is the key transcription factor mediating the GM-CSF or FLT3L induced up-regulation of CD11c in cDCs, because PU.1 knockdown markedly reduced CD11c activation in response to these cytokines. PU.1 knockdown demonstrated that PU.1 is also required for FLT3L induced CD11c expression in pDCs. GM-CSF also induced activation of CD11c in CD8^+^ cDCs, even though GM-CSF alone is not sufficient to promote the differentiation of CD8^+^ cDCs. Thus, the molecular mechanism on how CD11c gene is activated by GM-CSF signaling in CD8^+^ DCs remains to be elucidated. The findings that GM-CSF did not further activate CD11c expression in pDCs, and that GM-CSF induction or Pu.1 expression cannot promote CD8^+^ DC differentiation, indicate that different transcription factors are responsive to the same extracellular signaling in specific DC subsets, and that specific combinations of transcription factors contribute to terminal cell differentiation of these different DC subsets.

## Materials and Methods

### Mice, Bone Marrow Cell Culture and Retroviral Gene Transfer

CD11c-Cre mice (C57BL/6J-Tg(Itgax-cre,-EGFP)4097Ach/J) and Rosa26 loxP-STOP-loxP YFP mice (B6.129X1-Gt(ROSA)26Sortm1(EYFP)Cos/J) were purchased from The Jackson Laboratory (Bar Harbor, ME). To generate mice carrying both the transgenes, CD11c-Cre mice were bred with Rosa26 loxP-STOP-loxP mice and offspring pups were subject to genomic PCR for genotyping. Mice that carry one allele of the CD11c-Cre and one allele of the Rosa26 loxP-STOP-loxP were selected for subsequent analysis. Six to twelve week old mice were used for all studies. All animal studies were approved by the University of Massachusetts Institutional Animal Care and Utilization Committee.

For bone marrow cell culture, bone marrow cells were flushed from femora and pelves of indicated mice with RPMI (American Type Culture Collection [ATCC] Manassas, VA) plus 10% fetal bovine serum (FBS; Invitrogen, Carlsbad, California). NIH/3T3 and HEK293 (ATCC, Manassas, VA) cells were maintained in DMEM (Invitrogen Corp., Carlsbad, CA) with 10% FBS. Bone marrow cells were grown in RPMI/10% FBS with GM-CSF at 30 ng/mL, or FLT3L at 100 ng/mL for 6 or 11 days. GM-CSF and FLT3L were purchased from PeproTech Inc. (Rocky Hill, NJ). For in vivo administration, mice were intraperitoneally injected with GM-CSF at 100 ng/gram body weight and cells were collected 48 hours later for analysis. For retroviral shRNA transduction, bone marrow cells were transduced with retroviruses containing shRNA against Pu.1 or scrambled control (Open Biosystems Inc. Lafayette, CO) for 48 hours and grown in 2.5 µg ml^−1^ puromycin (Sigma-Aldrich, St. Louis, MO) for 3–5 days.

### Flow Cytometry Analysis and Immunoblotting

Lineage staining of bone marrow and spleen was performed with fluorescent conjugated antibodies against Gr1, CD11b, B220, CD3e, Ter119, F4/80 and Mac3 (eBiosciences, San Diego, CA). Other antibodies used for flow cytometry include: M-CSF-R, Ly6C, c-kit, Flt3, Sca-1, CD34 and FcγII/III (eBiosciences). Antibodies against CD11c, CD19 and CD49b were purchased from BD Biosciences (Franklin Lakes, NJ). Flow cytometry utilized LSRII or FACSAria II, respectively (BD Biosciences). Flow cytometry data were analyzed with Diva (BD Biosciences) and FlowJo software (Tree Star Inc, Ashland, OR). Immunoblotting was performed according to standard protocol. Antibodies against PU.1 and β-Actin were purchased from SantaCruz Biotechnology (Santa Cruz, CA).

### Reverse Transcription and Real-Time, Quantitative PCR

Reverse Transcription was performed with New England BioLabs DyNAmo cDNA synthesis kit, per manufacturer’s protocol, with an Applied Biosystems thermal-cycler. Quantitative Real-time PCR was performed with Qiagen real-time PCR master kit with an Eppendorf real-time thermal-cycler.

### Chromatin Immunoprecipitation (ChIP) Assays

Chromatin was immunoprecipitated with Pu.1 or Irf-4 antibody (Santa Cruz Biotechnology, Inc.), and protein G sepharose beads (Pierce Biotechnology, Inc. Rockford, IL). ChIP procedure was performed according to manufacturer’s protocol (Pierce Biotechnology). PCR primers for PU.1: 5′-ACAACTTCCCACCCTGACTG-3′, 5′-GGTTGTGAAGGTGTGGCTTT-3′; IRF-4: 5′-GCAGAGCAAGACCCTGTTTC-3′, 5′-CTGAGCATTGAAAGCAACCA-3′.
